# Estimation of fitness from energetics and life‐history data: An example using mussels

**DOI:** 10.1002/ece3.4004

**Published:** 2018-05-07

**Authors:** Kenneth P. Sebens, Gianluca Sarà, Emily Carrington

**Affiliations:** ^1^ Department of Biology and Friday Harbor Laboratories University of Washington Friday Harbor WA USA; ^2^ Dipartimento di Scienze della Terra e del Mare Università di Studi di Palermo Palermo Italy; ^3^ School of Aquatic and Fishery Sciences University of Washington Seattle WA USA

**Keywords:** climate change, energetics, fitness, intertidal, invertebrate, life‐history, mussels

## Abstract

Changing environments have the potential to alter the fitness of organisms through effects on components of fitness such as energy acquisition, metabolic cost, growth rate, survivorship, and reproductive output. Organisms, on the other hand, can alter aspects of their physiology and life histories through phenotypic plasticity as well as through genetic change in populations (selection). Researchers examining the effects of environmental variables frequently concentrate on individual components of fitness, although methods exist to combine these into a population level estimate of average fitness, as the per capita rate of population growth for a set of identical individuals with a particular set of traits. Recent advances in energetic modeling have provided excellent data on energy intake and costs leading to growth, reproduction, and other life‐history parameters; these in turn have consequences for survivorship at all life‐history stages, and thus for fitness. Components of fitness alone (performance measures) are useful in determining organism response to changing conditions, but are often not good predictors of fitness; they can differ in both form and magnitude, as demonstrated in our model. Here, we combine an energetics model for growth and allocation with a matrix model that calculates population growth rate for a group of individuals with a particular set of traits. We use intertidal mussels as an example, because data exist for some of the important energetic and life‐history parameters, and because there is a hypothesized energetic trade‐off between byssus production (affecting survivorship), and energy used for growth and reproduction. The model shows exactly how strong this trade‐off is in terms of overall fitness, and it illustrates conditions where fitness components are good predictors of actual fitness, and cases where they are not. In addition, the model is used to examine the effects of environmental change on this trade‐off and on both fitness and on individual fitness components.

## 1 INTRODUCTION

The capacity of organisms to respond to environmental variability, including unidirectional change, depends on phylogenetic, genetic, physiological, and developmental constraints, and such responses can be short term (acclimation, phenotypic plasticity) or long term (adaptation, selection). The overall success of any set of adaptations, including morphological, physiological, or life‐history traits, can best be assessed by examining their effects on fitness (Endler, 1986; Kozlowski, 1993) which can also be used as a measure of natural selection (Arnold & Wade, 1984a,b). Although it is common to assess short‐term fitness components (often termed “fitness” or “Darwinian fitness”), such as growth rate, energy balance, reproductive output per season, mortality rate, or lifetime reproduction (Thoday, 1953), it is more informative to include all such response variables within a multi‐generation measure of fitness (de Jong, 1994). For example, a fitness component such as growth rate may be high under one set of conditions, but this could lead to high mortality from predators and thus lower fitness. Individual fitness components (e.g., survivorship, competitive ability, offspring produced) are also now being used as organismal performance assays (OPAs) for drug effects (Gaukler et al., 2015) and have been used to estimate fitness as short‐term or lifetime reproductive success alone (e.g., Hämäläinen et al., 2017).

To estimate fitness, researchers have used combinations of empirical measurements and mathematical modeling to generate comparative measures of population growth rate (i.e., *r* or λ, where *r* = lnλ) (McGraw & Caswell, 1996; Metz, Nisbet, & Geritz, 1992) for individuals within a population, or for a hypothetical population of identical individuals (genotypes or phenotypes) with those traits (Buckley & Kingsolver, 2012b; Kozlowski, Czarnoleski, & Danko, 2004; Sebens, 2002). One general definition of fitness is “contribution of N fertile offspring to the next generation,” which does not consider the rate at which they are added, nor what happens in future generations. Using r as a fitness estimate incorporates both, and lambda can be considered the average contribution of new individuals per year (generation, or other time unit) over many generations. Comparing such fitness estimates for a range of traits under study provides a powerful prediction of how well certain phenotypes will perform under existing, new, or varying environmental conditions (Buckley & Kingsolver, 2012a; Kawecki & Stearns, 1993; Kozlowski, 1993; Lande & Arnold, 1983; Pelletier, Clutton‐Brock, Pemberton, Tuljapurkar, & Coulson, 2007) and how populations might respond to such changes (e.g., integral projection models: Merow et al., 2013; Rees, Childs, & Ellner, 2014; Elahi, Sebens, & De Leo, 2016).

There are two common approaches to estimating fitness in field populations. One is biophysical, where performance (e.g., egg production) is measured over a range of physical environmental conditions (e.g., temperatures); then, offspring production rates (e.g., as a function of body temperature) are used with survivorship to develop life tables and calculate fitness (e.g., as λ) (Buckley & Kingsolver, 2012a,b). A second approach uses energetics as a basis for estimating fitness (Sebens, 2002). Recently, there have been improvements in methodology and computational power applied to whole organism and population level energetics, including the dynamic energy budget (DEB) approach (Kooijman, 2010; Nisbet, Jusup, Klanjscek, & Pecquerie, 2012), which allows organismal energy balance, growth, and reproductive output to be determined for varying physical environments, over the lifetime of an individual. Realistic environmental conditions can be incorporated at fine temporal scales (e.g., hourly, Saraiva et al., 2012; Sarà, Palmeri, Montalto, Rinaldi, & Widdows, 2013; Matzelle, Montalto, Sarà, Zippay, & Helmuth, 2014; Montalto, Palmeri, Rinaldi, Kooijman, & Sarà, 2014; Montalto, Sarà, Ruti, Dell'Aquila, & Helmuth, 2014), to examine the effect of changing conditions on fitness components, or to examine the effect of changing organism traits under any spatiotemporal change of environmental conditions. However, there have been few attempts to combine the fitness estimation approach (McGraw & Caswell, 1996) based on life‐history data with the well‐established energy budget models (Kooijman, 2010; Nisbet et al., 2012; but see Sebens, 2002; Nisbet, McCauley, & Johnson, 2010).

It is often not possible to follow individuals over their lifetimes, but fertility, growth, and survivorship data might be available for limited time periods and for certain subsets of individuals. McGraw and Caswell (1996) suggested that a productive modeling approach would take the available limited empirical data and embed it in a model using simulated data for all other rates, with the model then able to show the effect of variable components on overall fitness. This is particularly important for marine invertebrates with open populations and long‐range dispersal, where some of the survivorship and recruitment data are nearly impossible to get. Our model uses this approach, incorporating existing data where possible and integrating model simulations where necessary. The intent in this effort is not to describe current population growth or determine whether existing populations will increase or decrease as conditions change, but instead to examine the effects of variable traits on fitness. With enough data from a field population, this approach can also be used to predict demographic shifts with climate change, impacts of harvesting and species introductions, or other factors that might impact growth, survivorship, and fecundity (as in Buckley & Kingsolver, 2012a,b). Furthermore, the model can incorporate high‐frequency change in environmental parameters, as in the dynamic energy budget approach (Sarà, Rinadi & Montalto, 2014). The effects of variable traits on fitness, as affected by environmental variability, can thus also be examined individually and under numerous scenarios.

Here we describe a modeling approach that combines energetic and life‐history information to investigate the effects (on fitness) of multiple physical drivers of energetic cost and intake, as well as differential allocation of resources to growth, metabolism, reproduction, and nonliving structures, using mussels as a model system. We developed this model for a generic small mussel, starting from the invasive mussel *Brachidontes pharaonis* model in the Mediterranean (Sarà, Porporato, Mangano, & Mieszkowska, 2018) for which published data were available for most of the energetic parameters (Sarà, Palmeri, Rinaldi, Montalto, & Helmuth, 2013). Where data were not available for this species, we used published information from other mussel species to test the model.

## 2 MATERIALS AND METHODS

For the energetics model, the basic growth equations from Sebens (1982, 1987, 2002) were used for growth, energy surplus (intake‐cost), and final asymptotic size (*M*_opt_), where energy surplus (*E*_s_) is equivalent to scope for growth (and reproduction). Allocation of energy to byssus production is incorporated as an increase in metabolic cost during byssus production (as in Lurman, Hilton, & Ragg, 2013). Production of other nonliving structures, such as shell, is assumed to be a fixed part of metabolic cost, only because we are not varying that allocation in the current formulation. Here, metabolic cost includes all energy used to construct, maintain, and replace all tissues including permanent gonadal structures, as well as production of mucus, shell, byssus, and other nonliving products and exudates. Eventually, a more complicated formulation could address shell deposition independently and include shell resorption and repair. Energy surplus, available for somatic growth and reproduction, is thus:

(1) $$E_{s}=aM^{c}-bM^{d}$$

where *E*_s_ is energy surplus (energy intake *aM* ^*c*^ minus cost *bM* ^*d*^), *a* and *b* are scalars, and *c* and *d* are exponents relating energy intake and metabolic cost to organism size (mass, *M*). For the model, we chose *c* = 0.67 and *d* = 1.0 based on Sarà, Palmeri, Montalto, et al. (2013) and Montalto, Palmeri, et al. (2014); in general, the value of c is lower than *d* (Sebens, 1987). Energy intake is often a function of feeding surface area, whereas cost is proportional to the 0.75 power of mass (Kleiber's Law) or to mass with exponents up to 1.0 (proportional to mass) in some organisms (Kooijman, 2010; Patterson, 1992; Sebens, 1987; West, Brown, & Enquist, 1997).

**Figure 1:**
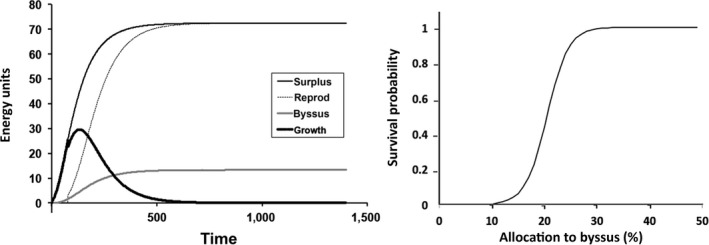
Left. Daily energy allocation (Joules per day) over time (days) during growth of an individual, calculated in daily time steps over a 4 year lifespan, with constant food availability and temperature. Right. Survival probability as a function of energy allocation to byssal thread production (multiplier of metabolic cost). Here, 20% represents a 20% increase in metabolic cost

The scalars *a* and *b* are derived empirically from functions for food availability, temperature, and other environmental factors, as well as from trait variation (e.g., schedule of allocation) and assimilation. The data for energy intake, cost, and allocation to reproduction used in this model are for *Brachidontes pharaonis* unless noted (from data in Sarà, Palmeri, Montalto, et al., 2013; Montalto, Palmeri, et al., 2014). In this model, *a* = food availability x feeding surface area × assimilation efficiency (Table 1) and is equivalent to ingestion rate (IR) multiplied by assimilation efficiency (Sarà, Palmeri, Montalto, et al., 2013). The scalar *b* is derived from literature values for small mussels (Sarà, Palmeri, Montalto, et al., 2013; Montalto, Palmeri, et al., 2014; Matzelle, Montalto, Sarà, Zippay, & Helmuth, 2014) (= 0.0145). Growth of somatic tissue occurs in each time step of the model (per day in this example) such that:

(2) $$\Delta M/\Delta t=e(E_{s}-E_{r})$$

where somatic mass is added based on energy surplus minus energy allocated to reproduction. At each time step, energy allocated to somatic tissue growth, reproductive tissue growth and gametes, metabolic cost, and nonliving structures is summed and graphed (Figure 1). The scalar *e* converts energy units to tissue mass (maximum storage density, Table 1). In this model, growth ceases as *M*_opt_ is reached, and all energy goes to reproduction. Note that, with size‐dependent mortality rates, it can be adaptive to grow beyond that size (Sebens, 2002), or to stop growth below that size, and it is thus an optimum only in energetic (not fitness) terms. Growth rate at any time unit is calculated as the energy used for growth (from surplus, expressed as mass equivalent), divided by the somatic mass at that time, per time unit. We chose 300 mg as a size to compare early growth rates because this is just below the size at maturity. Reproduction does not occur below this threshold mass in this model, and above that is allocated as an increasing percentage (0–100%, linear) of the surplus until growth stops at the optimal mass, *M*_opt_. This is the simplest allocation function, although real ones could be strongly nonlinear.

**Table 1:** Parameters used in the model, with their range of values and data source

Parameter	Units	Values (model)	Values (source)	Source
Ingestion rate (*I*)	J h^−1^ cm^−2^	18	17.88 ± 14.30^a^	Sarà, Palmeri, Montalto, et al. (2013)
Maintenance costs (*R*)	J h^−1^ g^−1^	14	14	Montalto, Palmeri, et al. (2014) and Montalto, Sarà, et al. (2014)
Maximum storage density	J g^−1^	1,967	1967 ± 190 J/cm^3^	Sarà, Palmeri, Montalto, et al. (2013)
Mass at birth (recruit)	g	0.0000005	0.00000049 cm^3^	Sarà, Palmeri, Montalto, et al. (2013)
Mass at sexual maturity	g	0.01008	0.01008 cm^3^	Sarà, Palmeri, Montalto, et al. (2013)
Assimilation efficiency (AE)	None	0.75	0.75 ± 0.12	Conover (1966)
Arrhenius temperature (TA)	°K	8,232	8,232 ± 2,923	Sarà, Palmeri, Montalto, et al. (2013)
Reference temperature *T*_ref_	°K	285	293	Sarà, Palmeri, Montalto, et al. (2013)
Upper tolerance temperature (*T*_H_ *k*)	°K	298	None	This paper
Critical temperature (feeding)	°K	289	None	This paper
Temperature multiplier functions T_i_, T_c_	None	0.5–2.1	From equation	Sarà, Palmeri, Montalto, et al. (2013)^b^
Scalar a for intake	J d^−1 ^cm^−2^	var	*I* × 0.75	Sarà, Palmeri, Montalto, et al. (2013)
Scalar b for metabolic cost	J d^−1 ^cm^−3^	var	*R* × *T*	Sarà, Palmeri, Montalto, et al. (2013)
Exponent *c*	None	0.67	0.67	Sarà, Palmeri, Montalto, et al. (2013)
Exponent *d*	None	1.00	1.00	Sarà, Palmeri, Montalto, et al. (2013)
Eggs per individual per month	mo^−1^	571,578	811,700	Sebens et al. (2016)^c^
Eggs per joule allocation	eggs J^−1^	526	526	Sarà, Palmeri, Montalto, et al. (2013)
Survivorship egg to settler (*l*_*x*_)	mo^−1^	0.00013		This paper^d^
Survivorship settler to recruit (*l*_*x*_)	mo^−1^	0.01		This paper^d^
Survivorship, monthly (*l*_*x*_)	mo^−1^	0.90		This paper^b^^,^ d

a Maximum surface area‐specific ingestion rate; J h^−1^ cm^−2^.

b This maximum value is reduced when mussels allocate less energy to byssal threads. From Arrhenius equation, multiplier used to increase intake rate and/or metabolic cost over a specified range.

c Maximum value from model, and for *M. galloprovincialis*, averaged over 4 years (Sarà, in Sebens et al., 2016).

d Values chosen to provide stable population, *r* = 0 (data not available for field population).

Temperature effects on metabolic cost, and on energy intake, are modeled following Sarà, Palmeri, Montalto, et al. (2013) using the Arrhenius equation, but allowing for different degrees of influence on each process (as in Strong & Daborn, 1980). While there is often a clear (increasing) effect of temperature on metabolic rate, the effect on energy intake is less predictable and differs radically among phyla, and probably even among related species. For example, movement and digestion are affected by temperature, and thus, energy intake might increase with temperature up to some optimum and then decrease as temperature becomes stressful (e.g., sea stars, Sanford, 2000, 2002). For this model, we assumed that temperature effects are somewhat greater on metabolic cost (*T*_c_) than on energy intake (*T*_i_), and that energy intake increases with temperature to some maximum (critical temperature, Table 1), then decreases linearly (not by Arrhenius equation). This is based on the well‐known effect of temperature increasing metabolic rate in poikilotherms (i.e., **Q**_10_ relationship; Kooijman, 2010) and the few available data on temperature effects on food acquisition and energy intake in marine invertebrates. In passive suspension feeders, where intake depends on the surface area presented to moving water, there could be a range of temperatures at which temperature has little or no effect on capture (but may affect digestion or assimilation). Active suspension feeders often increase pumping rates with temperature (and food supply), over a certain temperature range, and this will also be reflected in increased metabolic cost. Temperature parameters and values used are given in Table 1. The multiplier functions T_i_ (t) and T_c_ (t) are used to modify intake and cost as:

(3) $$E_{s}=T_{i}\left(t\right)aM^{c}-T_{c}\left(t\right)gbM^{d}$$

where t is temperature, and g is allocation to byssus.

The life‐history model, an age structured Leslie Matrix (Leslie, 1945, 1948) Caswell (1989ab) based on that of Sebens (2002) and McGraw and Caswell (1996), calculates a per capita (exponential) growth rate (*r*) that can be used to estimate multi‐generation (overlapping generations) fitness for a population of identical (genotype, phenotype, or trait group) individuals (usually for females only, empirical sex ratio used to calculate males). This is the rate at which new individuals would be added to the population each time period (or generation, if non‐overlapping) if all individuals had the same relevant traits. McGraw and Caswell (1996) define fitness on a per individual basis (one matrix per individual in the population) but caution that this has drawbacks as not all identical individuals will have the same history even in a constant environment. We conceptualize our model population as a group of individuals all with the same traits (phenotypic expression), experiencing a particular environment (constant, or variable) over a prolonged time period during which the population reaches a stable age distribution and does not experience crowding (thus continued exponential growth). This definition allows us to examine what would happen to fitness if there were small differences in energy allocation, timing of reproduction, or any other energetic or life‐history trait. Note that this population should be considered embedded in a larger population of individuals with different traits, as in McGraw and Caswell (1996). Our formulation of the age‐structured Leslie Matrix, incorporating age‐specific fecundities in the top row and age‐specific survivorship on the diagonal, is identical to that used by McGraw and Caswell (1996) and Sebens (2002), with monthly increments of age over a 4‐year life span for these mussels.

In this instance, the model is not being used to predict future (real) population sizes, only to provide a comparison among alternative trait values. Thus, the criticism that populations are rarely in exponential growth phase, and thus traits do not evolve under those conditions (Kozlowski, 1999), does not hold. This method of estimating fitness relies on the “propensity” interpretation (McGraw & Caswell, 1996; Mills & Beatty, 1979) for a group of identical individuals, with the understanding that the specific life events of each individual could be very different even in the same environment, based on probabilities of survival, prey encounter, successful fertilization, and many other stochastic events. Theoretical life‐history studies use the population growth rate of a clonal, asexually reproducing, population as a simplification (same genotype, no sex) to examine variation in particular traits (Cole, 1954; Stearns, 1992; Stearns & Crandall, 1981) as they affect predicted fitness. Lenski and Service (1982) note that the mean lambda for a population (calculated per individual) is not equal to the finite rate of increase in the population under study, although McGraw and Caswell (1996) argue that this bias does not negate use of this method for comparative purposes. In our case, we are using the actual population growth rate after it attains a stable age distribution, not a mean rate per individual based on individual life tables. This method should be appropriate even under conditions of variable or changing environments where calculation of lambda as the dominant eigenvalue of the Leslie Matrix is not possible because the matrix elements themselves are constantly changing. We note that this type of model can also incorporate density dependence and can be used to examine the effects of particular traits under different degrees of crowding, for example (as in Nisbet et al., 2010), and might thus be used for longer term population projection.

In this study, as in many others, we do not have all the population parameter data needed to calculate actual population growth. Although mussel population densities vary from year to year at any one intertidal location, unless they are invading or going locally extinct, their long‐term population growth (decades) can be assumed to be near zero (i.e., populations are roughly stable). For our model, mortality rates of larvae, juveniles, and later size classes were chosen to make this so, in the absence of sufficient field data (following McGraw & Caswell, 1996). In fact, it is nearly impossible to get good measures of these rates in an open population with long‐distance dispersal. Therefore, we assume that, at some mean ambient set of conditions (example here, constant 16°C), the population has been stable for a long time period (*r* = 0, lambda = 1) and we use this as our reference condition for comparisons of variable traits or new environmental conditions. Based on maximum age measurements of about 4 years for small mussels, survival probability was set at 90% per month (such that less than 1% survive 48 months). The final missing information was survivorship from released egg to settled juvenile, which is a very small and variable number. To achieve a stable population (*r* = 0), this survivorship was determined to be 1.3 × 10^−6^, which, when multiplied by the initial number of eggs produced, provides the number of recruiting juveniles in each time step. This includes loss of eggs that are not fertilized, larvae lost by advection and predation, and mortality of settled larvae before they reach a size we define as a new recruit. This value was chosen to provide a stable population for the model.

To explore the effects of producing nonliving materials on fitness, we chose to examine byssal thread production. Mussel byssus is an assemblage of numerous collagenous fibers that tether these bivalve mollusks to hard substrates (e.g., Babarro & Carrington, 2013; Bell & Gosline, 1997; Carrington, Moeser, Dimond, Mello, & Boller, 2009; O'Donnell, George, & Carrington, 2013; Zardi, McQuaid, & Nicastro, 2007). Byssal thread quality and quantity affect a mussel's tenacity (Bell & Gosline, 1996; Carrington, 2002a,b; Moeser, Leba, & Carrington, 2006a,b) so increased production of strong threads will increase survivorship and reductions in thread quality and or quantity are likely to result in wave‐induced dislodgment or successful predation (Babarro & Carrington, 2013; Bell & Gosline, 1997; Carrington et al., 2009; Lachance, Myrand, Tremblay, Koutitonsky, & Carrington, 2008; Zardi et al., 2007). These factors create an obvious energetic trade‐off; energy that could have gone to somatic growth or reproduction must instead be used to produce enough byssus to ensure survival (Carrington, 2002a,b). We incorporated this trade‐off into our model to investigate its resulting impact on fitness. A range of byssus production costs, both lower and higher than those measured by Lurman et al. (2013), was used in the model (multiplier *g* in Eq. 3, above), as no comparable information was available for *Brachidontes*. Mortality due to byssus production was modeled as a logistic function (Figure 1) such that low production resulted in high risk of mortality (up to 100%) and high production reached a plateau where mortality rate was constant, and there was no effect of byssus allocation. In the intermediate region, producing more byssus results in higher survivorship. The equation used was as follows:

(4) $$S=1/(1+e^{-g})$$

where *S* is survival probability (per month) and *g* is the energy allocation to byssus, expressed as a fractional increase in metabolic rate, from 0% to 50% (*g* = 0–0.5). From 0% to 10% survival is zero, and above 30% it is 1, so most of the effect on survival is in the 10%–30% range. An allocation below 15, for example, would certainly result in a declining population, whereas an allocation of 40 would result in lower fitness due to lost opportunity for growth and reproduction. The exact relationship between byssus production and survivorship is not known for the species used in this model, but has been estimated for *Mytilus edulis* (Carrington et al., 2009).

For each set of conditions, the Leslie Matrix was run for 30 years starting with an arbitrary 10 individuals; a stable age distribution was achieved during the first 10 years or less. The matrix used 48 age categories (months), values in the top row were *l*_*x*_ *m*_*x*,_ where *l*_*x*_ is survivorship to age *x*, and *m*_*x*_ is fecundity at age *x*. The diagonal values are *m*_*x*_, and all other entries are 0 (as in Sebens, 2002). The slope of the natural logarithm of population size versus time was used to calculate *r*, the per capita rate of increase in the population (assuming all females). This slope was determined only for the final 6 years when it was certain the population was at a stable age distribution, following the method of Sebens (2002). This method allows for changes in any parameters among years; it does not depend on having the same matrix values over time. Alternatively, for any one set of invariable conditions, r can be determined as the dominant eigenvalue of the Leslie Matrix. Both methods were tested here and provided identical results.

## 3 RESULTS

**Figure 2:**
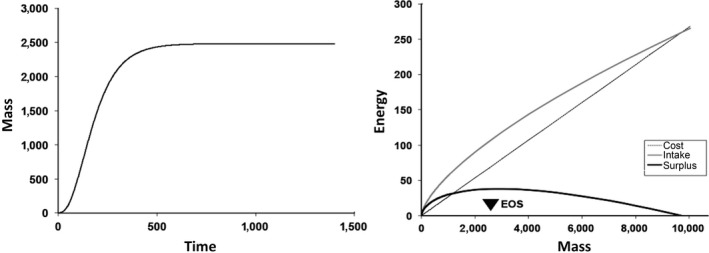
Left. Somatic growth of an individual (mass, mg), calculated over time (days), in daily time steps over a 4 year lifespan, with constant food availability and temperature. Right. Energy (Joules/day) intake, metabolic cost, and energy surplus for mussels over a range of sizes (mass, mg). When energy surplus is at a maximum in this model (at EOS), somatic growth stops. Above this mass, energy surplus declines and there is less energy available for reproduction

The allocation of energy to somatic growth, reproduction, byssus, and energetic surplus (intake‐metabolic cost and byssus) for one of the model runs is given in Figure 1. Note that energy used for growth peaks just below 200 days and then declines gradually to zero as the individual approaches a size asymptote at the energetic optimum (greatest surplus at *M*_opt_). This energetic surplus, or scope for growth (and reproduction), is used to produce somatic tissue, reproductive tissue, and gametes. Production costs of byssus, shell, and other nonliving structures and products in this model are subsumed in metabolic cost, although they could alternatively be allocated from the energetic surplus. The summed lifetime allocations to each compartment are illustrated in Figure 2, followed by the energy per time unit, as energy intake, energetic cost, and their difference (surplus). Note that the maximum surplus occurs at the point labeled EOS (Energetically Optimum Size) in this graph, and growth is predicted to stop at this point. Projecting to masses above this optimum, surplus would decline to zero at some theoretical maximum size where neither growth nor reproduction could occur (Sebens, 1982, 1987); there are, of course, situations where fitness would be maximized at sizes either below or above the predicted energetic optimum (Denny, 1988; Sebens, 2002) and thus the EOS could be exceeded, or never reached. Our combined models can be used to predict a different optimum size, based on maximizing fitness (OS) (Optimum Size, based on fitness, including energetics), which could then be compared to the EOS to determine which factors (energy, mortality) are setting the maximum sizes observed in a population. In this study, we consider only the case where at least some individuals in the population reach the EOS. In a previous study, Sebens (2002) examined the conditions that favored earlier reproduction, and an OS smaller than the EOS.

The model was run for a range of temperatures (5–20°C), where intake and cost both relate to temperature by the same Arrhenius equation, up to a critical temperature (chosen as 16°C) after which cost continues to increase, but intake decreases linearly to zero at some maximum temperature (25°C here). Although we do not know what the critical temperature for maximizing intake is for this species, this value is well within the range tested by Sarà, Palmeri, Rinaldi, et al. (2013) and well below the upper lethal temperature of 32°C for *Brachidontes pharaonis*.

### 3.1 Components of fitness

**Figure 3:**
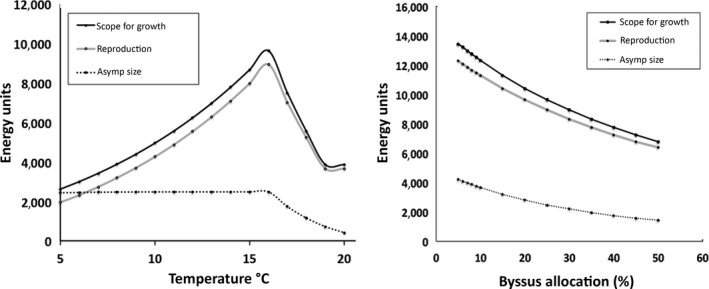
Lifetime energy allocation (Joules), summed scope for growth, summed reproduction, asymptotic size, (left) for a range of temperatures and (right) for a range of rates of byssus production (as percent increase in metabolic rate)

**Figure 4:**
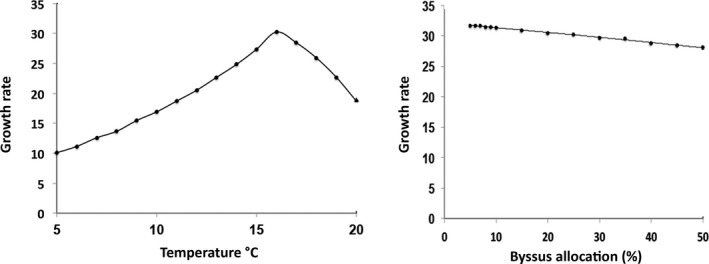
Growth rate (mg per day) just before reproductive maturity, when allocation of energy to growth is at a maximum (left) for a range of temperatures and (right) for a range of rates of byssus production (as percent increase in metabolic rate

The lifetime energy surplus (scope for growth) and lifetime reproductive output have the same directional response, as expected, but asymptotic size does not change much, especially in the low temperature region (Figure 3). The model was also run for a range of byssus production rates (Figure 3) that represents a 5%–50% increase in metabolic rate averaged over all time periods. This sensitivity analysis shows how components of fitness react to changes in allocation of energy to byssus production, compared to how they react to temperature alone. These results show that lifetime scope for growth, lifetime reproductive output, and asymptotic size all decrease nonlinearly with increased allocation to byssus, as expected. Growth rate just before reproductive maturity, when percent allocation of energy to growth is at a maximum, also decreased but did not change as much over a broad range of allocation of energy to byssus and thus, like asymptotic size, would be a poor predictor of fitness or capacity for population change for this mussel (Figure 4).

All of the individual components of fitness are frequently used to compare performance across populations. In this example, measuring asymptotic size alone, or growth rate, would produce a very different picture of habitat suitability, temperature optima, or expected population performance. Although such components of fitness are valid measures of performance, they are not enough in themselves, to predict overall fitness or population response. These results also suggest that direct measurement of energetic components (scope for growth) or reproductive output are more sensitive measures of performance than are growth rate or size alone, as their magnitude varied more for the same range of environmental parameters.

### 3.2 Estimates of fitness

**Figure 5:**
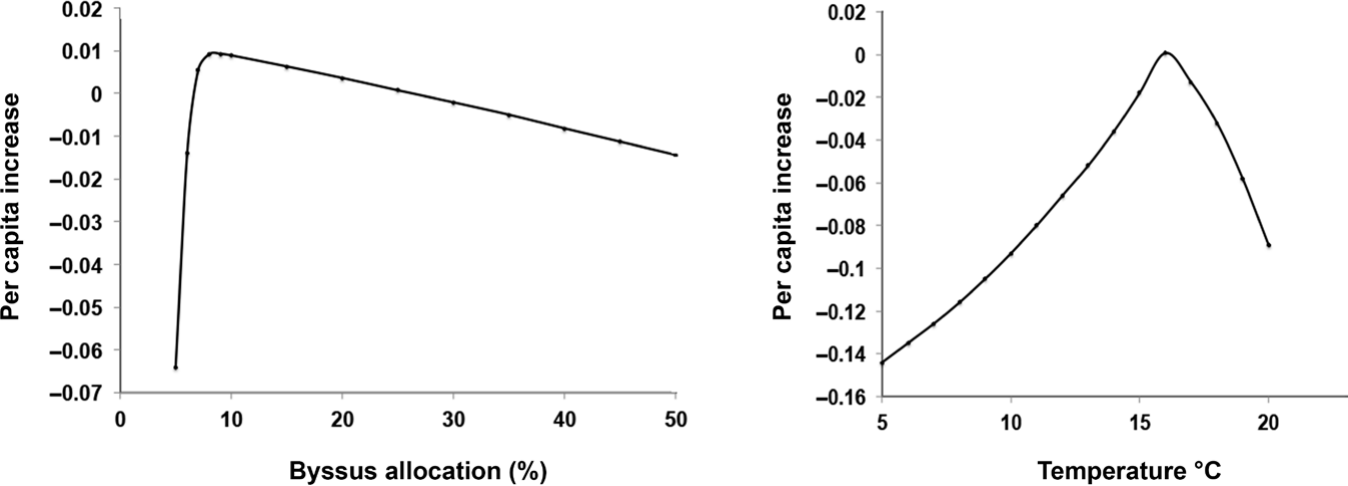
Fitness (*r* = per capita rate of increase) over a range of mean temperatures (right) and for a range of rates of byssus production (as percent increase in metabolic rate) (left). Note that the shape of the response curve for fitness (*r*) is very different than for the other components of fitness (Figures 3 and 4) considering byssus allocation

Using this model, the estimate of average fitness (*r*) for a range of mean temperatures was similar to that for two of the fitness components, scope for growth, and lifetime reproductive output, but was not similar to that for asymptotic size or early growth rate as responses to temperature (Figure 5). For byssus allocation, however, the fitness response was very different than the response for several measures of performance, with a decrease in fitness at both high and low allocations. At the high end, this is due to loss of energy that could have been allocated to growth and reproduction. At the low end, mortality increases when byssus production is insufficient and mussels are easily dislodged. Thus, the overall effect on either fitness or population growth, of any environmental parameter or life‐history trait, may not be accurately represented by single fitness components (performance) and must be assessed using the entire life table to generate a fitness estimate (e.g., as population growth rate).

## 4 DISCUSSION

The combined energetics and life‐history approach illustrated here provides an estimate of average fitness for any one genotype or phenotype, or for a group of individuals sharing the same traits. This fitness measure has been favored in several previous studies of field populations (Buckley & Kingsolver, 2012a,b; Fodrey, Levin, & Lucas, 2009; Pelletier et al., 2007) because it sums up the effects of multiple factors that affect growth, fecundity, and survivorship (fitness components). Factors affecting fitness can be environmental parameters, such as temperature or pH, or internal energy allocations such as to byssus production (this study), or any other life‐history trait, such as age at first reproduction (McGraw & Caswell, 1996). Components of fitness alone (performance measures) are useful in determining organism responses to changing conditions, but are often not good predictors of fitness or population change; they can differ in both form and magnitude, as for mussels in this model (Figures 3, 4, 5). Mortality of mussels due to low byssus production, a certainty in field situations with heavy wave action (Carrington et al., 2009), causes fitness measures to deviate widely from energetic or performance measures that do not incorporate survivorship. McGraw and Caswell presented a similar case (for sparrow hawks) where a component of fitness (lifetime reproductive output) was not affected by age at first reproduction, whereas fitness (as lambda) was significantly reduced by increasing age at first reproduction. Such examples may be very common. Consider the hypothetical scenario where more food results in a higher growth rate and reproductive output, but their larger size makes individuals susceptible to predators earlier (or even attracts them). Habitats that are energetically less good could actually have higher average fitness due to underexposure of larger individuals to predation. In such cases, the average fitness will provide a much different picture than would scope for growth, or growth rate alone. The coupled approach used here allows us to examine changes in environmental parameters that affect energetics, and also determine how changes in survivorship affect overall fitness.

In this study, we describe a modeling approach and give an example of how it can be used for intertidal mussels. There is a wealth of energetics and life‐history data from mussel populations in diverse regions (Fly & Hilbish, 2013; Grant, 1996; Grant & Bacher, 1998; Matzelle et al., 2014; Melzner et al., 2011; Sarà, Palmeri, Montalto, et al., 2013; Sarà, Palmeri, Rinaldi, et al., 2013) that can be used for further analysis. We also incorporated information on byssus, the fibers that tether these bivalve mollusks to hard substrates (Carrington and Gosline 2002ab). Whole animal byssus strength, or tenacity, cycles seasonally and causes wild and commercially farmed populations to “fall‐off” or dislodge during specific times of the year. *Mytilus edulis* in the Northwest Atlantic coast of the USA and Canada are prone to dislodgement in late summer and early fall, when weak attachment coincides with increased storms or harvest activities (Carrington et al., 2009; Lachance et al., 2008). In contrast, the same species on the Atlantic coast of the UK is at risk of dislodgment in late winter and early spring because weak attachment occurs 4 months earlier (Carrington, 2002b; Price, 1980). Dislodgment results in mortality, and thus, byssus production is a major factor determining survivorship over the mussel's lifetime. Both temperature and pH (or pCO_2_) affect byssus properties and attachment to surfaces; they fail more readily, with less force applied, at higher temperatures and lower pH (O'Donnell et al., 2013).

In this model, there is a clear trade‐off between energy for byssus production (enhanced survival) and energy for growth, maintenance, and reproduction. The combination of a life‐history perspective, with both physiological (Denny & Helmuth, 2009) and biophysical approaches (Helmuth, Kingsolver, & Carrington, 2005), can be a very powerful method to examine these trade‐offs as they affect fitness. Models of this type can be used to examine trait variation in field or theoretical populations. For well‐studied mussel species, for example, we can examine the energetic costs of building tissue and nonliving structures (shell, byssal threads) when produced over a range of physical conditions, and when conditions vary (daily, seasonally, spatially; e.g., Elliott et al., 2008) as they do in many habitats. We can also examine how allocation of energy among multiple compartments affects fitness over a range of conditions. Increased or decreased allocation to growth and reproduction at different stages in the life cycle would be one example and differential allocation to shell production and byssal thread production are others. Effects on survivorship (e.g., having more shell, more byssus) will be incorporated, allowing us to measure the trade‐offs between these allocations, in terms of fitness.

Increasing temperatures are predicted to affect populations of ectotherms in multiple ways, including through energy allocation (Daufresne, Lengfellner, & Sommer, 2009; Forster, Hirst, & Atkinson, 2012; Forster, Hirst, & Woodward, 2011; Sheridan & Bickford, 2011; Zuo, Moses, West, Hou, & Brown, 2012) and organism size (Angilletta & Dunham, 2003; Angilletta, Steury, & Sears, 2004; Frazier, Huey, & Berrigan, 2006; Kingsolver & Huey, 2008). Although we used temperature as a prime environmental parameter in this model, other aspects of the marine environment are also changing rapidly and can affect fitness of mussels and many other organisms. Ocean temperature is increasing globally, and acidification is occurring at a rate faster than has been experienced on the planet for at least the last 50 million years. A clear understanding of the ocean's carbonate system is emerging and is essential to predictions of the organism‐level feedbacks and impacts to be expected as a result of future increases of anthropogenic pCO_2_ (Dickson, 2012; Doney et al., 2012; Feely, Sabine, Hernandez‐Ayon, Ianson, & Hales, 2008; Feely et al., 2010; Hoegh Guldberg and Bruno 2008; Hoegh‐Guldberg, 2012). In many coastal regions, effects of climate change are already evident (Crim, Sunday, & Harley, 2011; Gaylord et al., 2011; Gilman, Urban, Tewksbury, Gilchrist, & Holt, 2010; Helmuth et al., 2010; Wootton & Pfister, 2012), and some locations have experienced low pH conditions long enough for local adaptation to have occurred already (Murray et al., 2015). These global drivers can interact with local change in environmental conditions (e.g., hypoxia events; Sarà, Mangano, Johnson, & Mazzola, 2018) complicating the situation, and highlighting the necessity of investigating both mechanical properties and life‐history characteristics to forecast future effects on local biodiversity. Indeed, ecologically important species (e.g., keystone species, foundation species, ecosystem engineers) will be impacted by environmental change, causing unforeseen and often undesirable changes in community composition and species diversity (Maas, Wishner, & Seibel, 2012; Menge, 2012; O'Donnell et al., 2013; Sarà, Milanese, et al., 2014; Wethey et al., 2011). Some commercially important species will also be impacted (e.g., oysters, mussels, clams), influencing harvest (Sarà, Mangano, et al., 2018), and thus, it is critical to have both empirical data and models that describe and predict the effects of changing ocean temperature and chemistry at both the individual (DEB) and population (fitness, population projection) scales. The response of individual organisms can be studied in the laboratory, but the full population and community response can only be studied in the natural environment (Buckley & Kingsolver, 2012b). The approach presented in this study, combining an energetics model and a life‐history model, is a preliminary step in that direction. Such models can incorporate multiple responses to environmental parameters, as well as internal allocation of energy to growth, reproduction, and to nonliving structures that influence survival (e.g., byssus, shell). As a next step, the availability of advanced DEB models that can incorporate multiple environmental factors changing at high frequency, as well as complex internal allocation of energy, will provide further important information that can be used to increase the realism and utility of fitness models of this type.

## CONFLICTS OF INTEREST

None declared.

## AUTHOR CONTRIBUTION

KPS developed the mathematical model used in this research. EC and GS provided data and formulated methods used in the research. All authors contributed to writing and editing the manuscript.

## DATA ACCESSIBILITY

All data used in this manuscript are from published papers, as referenced.
